# Falling Victim to Wasps in the Air: A Fate Driven by Prey Flight Morphology?

**DOI:** 10.1371/journal.pone.0152256

**Published:** 2016-04-05

**Authors:** Yolanda Ballesteros, Carlo Polidori, José Tormos, Laura Baños-Picón, Josep D. Asís

**Affiliations:** 1 Área de Zoología, Facultad de Biología, Universidad de Salamanca, Salamanca, Spain; 2 Instituto de Ciencias Ambientales (ICAM), Universidad de Castilla-La Mancha, Avenida Carlos III, s/n, E-45071, Toledo, Spain; 3 Centre for Environmental and Marine Studies (CESAM), Departamento de Biologia Animal, Faculdade de Ciências da Universidade de Lisboa, C2-P3 Campo Grande, 1749–016, Lisboa, Portugal; USDA-Agricultural Research Service, UNITED STATES

## Abstract

In prey-predator systems where the interacting individuals are both fliers, the flight performance of both participants heavily influences the probability of success of the predator (the prey is captured) and of the prey (the predator is avoided). While the flight morphology (an estimate of flight performance) of predatory wasps has rarely been addressed as a factor that may contribute to explain prey use, how the flight morphology of potential prey influences the output of predator-prey encounters has not been studied. Here, we hypothesized that flight morphology associated with flight ability (flight muscle mass to body mass ratio (*FMR*) and body mass to wing area ratio (wing loading, *WL*)) of Diptera affect their probability of being captured by specialized Diptera-hunting wasps (*Bembix merceti* and *B*. *zonata*), predicting a better manoeuvrability and acceleration capacity achieved by higher *FMR* and lower *WL*, and flight speed achieved by higher *WL*. In addition, wasp species with better flight morphology should be less limited by an advantageous Diptera flight morphology. Overall, the abundance of dipterans in the environment explained an important part of the observed variance in prey capture rate. However, it was not the only factor shaping prey capture. First, higher prey abundance was associated with greater capture rate for one species (*B*. *merceti*), although not for the other one. Second, the interaction observed between the environmental dipteran availability and dipteran *WL* for *B*. *zonata* suggests that greater dipteran *WL* (this probably meaning high cruising speed) decreased the probability of being captured, as long as fly abundance was high in the environment. Third, greater dipteran *FMR* (which likely means high manoeuvrability and acceleration capacity) helped to reduce predation by *B*. *merceti* if, again, dipterans were abundant in the environment. Wasp *WL* only varied with body mass but not between species, thereby hardly accounting for inter-specific differences in the wasps’ predatory patterns. However, the greater *FMR* of *B*. *zonata*, which implies better flight performance and greater load-lifting capacity, may explain why the capture rate in the two wasp species is affected by different factor interactions. In conclusion, although prey availability remains the primary factor shaping prey use, prey flight morphology seems to gain an additional role under conditions of abundant prey, when wasps can avoid flies with better flight ability.

## Introduction

Predation is a heavy driving force for the evolution of morphological and physiological traits [1]. Prey and predators are contestants in a continuous arms race on an evolutionary timescale, owing to the implications that success by either party may have on each other’s fitness [2]. However, prey-predator encounters are asymmetric interactions, as a failure by the predator means a missed meal, whereas a failure by the prey results in death. Thus, the ability of the prey to escape the predators should have become evolutionarily actively involved in the predator’s success or failure, in such a way that their own morphological features, apart from those of the predators, are decisive in determining the final result of the predation events. Combes *et al*. [1] have already remarked that beside the study of the active prey choice of predators, assessing the prey characteristics, such as their behaviour, biomechanics and ecology, is essential for addressing prey-predator interactions in a proper, non-fragmentary fashion. The existing background in the study of prey-predator interactions shows how much prominence has traditionally been given to predators’ attributes (for example, foraging behaviour and hunting-related morphology) and to the “passive” traits of the prey, such as their size, the odour enabling their localization by the predators, the microhabitats inhabited and the temporal prey-predator overlap [3–16]. In contrast, less often has the success or failure during a predation event been approached as dependent on the ability of the prey to actively evade a predator’s attack [1, 17–19]. Previous studies, mainly on birds, have highlighted the large extent to which prey behaviour and morphology contribute to the outcome of predator-prey interactions, ultimately shaping the diet of predators [20–27]. Insects include important groups of predatory animals for which similar mechanisms could operate, and thus deserve a deeper research in this respect.

One putative trait of flying insect prey that could be analysed within this framework is the morphology of the body parts related to flight. Flight-associated morphology influences different aspects of flight activity, such as speed, manoeuvrability (which generally indicates the ability to change the speed and direction of the movement [5, 28]) and load-lifting capacity (i.e. the maximum load that could be carried in flight) [18, 29–35]. The relationship between morphology and flight capacity may sometimes be difficult to interpret: for example, the influence that morphology has on the mentioned aspects may also be subject to the “type” of flight performed (clap-and-fling *versus* conventional wingbeat fliers, see [29]). Speed and manoeuvrability are, in turn, involved in the chance of the prey escaping a predator, and/or in the predator’s ability to hunt a prey [15–17, 26, 36].

Flight performance may be estimated through different indices that take into account morphological features; amongst them, the flight muscle ratio (ratio of flight muscle mass to body mass, *FMR*) and the wing loading (ratio of body mass to wing area, *WL*) have been empirically recognized as two of the most accurate for conventional wingbeat fliers [29], which are the subjects studied in the present work. These morphological features have been used consistently in ecological studies involving indirect evaluation of flight ability [14, 16, 29, 37, 38]. The *FMR* accounts for 99% of the load-lifting capacity of an animal [29], and positively affects acceleration and the ability to perform rapid changes in speed and flight direction [36, 39–42]. For their part, *WL* has been reported to have complex effects on flight ability. From one side, animals with relatively large wings compared to body size enjoy proficient manoeuvrability [21, 43], can fly more efficiently (in energy-saving terms) [41, 43, 44], and are able to take off at higher speeds [45], such patterns having been observed in butterflies, birds and bats [21, 27, 41, 43–45]. On the other side, *WL* is positively correlated with cruising flight speed, a pattern detected in butterflies [17, 36, 45, 46].

In line with the predator-biased approach generally carried out in the study of the prey-predator systems in insects, in central-place hunting wasps (Hymenoptera: Apoidea and Vespoidea), wasp flight morphology, together with wasp body mass, have been previously assessed to try to explain why some prey species are abundantly hunted, while others are rarely represented or even absent in their prey spectra. For example, at the individual level, greater *FMR* values and body masses are known to allow females to transport larger prey to the nest, whereas lower values of both parameters prevent wasps from including heavy prey in the diet of their larvae [47–52]. Nevertheless, whether the flight morphology of the potential prey also affects the probability of being hunted by the wasps still remains unknown.

In the present study, we aim to assess this topic using two hunting wasp species (*Bembix merceti* Parker, 1929 and *Bembix zonata* Klug, 1835 (Hymenoptera: Crabronidae)), and their only prey (Diptera) as models. Despite the fact that the Iberian sand wasps of the genus *Bembix* restrict their prey to flies, they are considered to a large extent plastic or opportunistic regarding their prey use, in such a way that they have been observed to exploit different dipteran species, depending on the year or population studied [15, 53]. The environmental availability of the different fly species in the neighbourhood of the wasps’ nesting area has been shown to be extremely important in shaping prey use in digger wasps; nevertheless, it has been observed that divergences between the available and the captured prey may occur, in such a way that some potential prey species can be overhunted (i.e. captured at frequencies higher than those expected from their availability), and others almost ignored despite their great availability [11, 12, 15, 53]. Prey body mass partially accounts for this deviation from opportunism [11, 12, 15], but still an important portion of variance remains unexplained.

We hypothesize that the flight morphology of the prey may also account for prey use. Specifically, based on the predictions of flight performance (estimated from flight morphology), we hypothesize: 1) that greater *FMR* and lower *WL* values are associated with dipterans little represented among the prey, because they presumably have a greater escape capability or are more difficult to capture; and 2) that the wasp species with greater *FMR* and lower *WL* is less affected by the flight morphology of the flies, and thus will be able to successfully catch dipterans with higher *FMR* and lower *WL* values.

## Materials and Methods

### Prey-predator systems and study area

The Mediterranean predatory digger wasps *B*. *merceti* and *B*. *zonata* are central-place foragers [16], that travel from the nest to different resource patches to get provisions for their larvae, going back to the nest with a single prey per trip. Previous research has reported many species of flies belonging to up to eight families (Anthomyiidae, Asilidae, Bombyliidae, Calliphoridae, Muscidae, Syrphidae, Tabanidae and Therevidae, in the case of *B*. *zonata*; Bombyliidae, Calliphoridae, Muscidae, Sarcophagidae, Stratiomyidae, Syrphidae, Tabanidae and Tachinidae, in the case of *B*. *merceti*) as prey of these wasps [15, 54–60].

The study was carried out in June-August of 2009, 2010 and 2011 in the neighbourhood of Almarail (province of Soria, NE Spain), in a siliceous and sandy area covered with sparse shrubby vegetation, largely shared by the females of both predator species (see Asís *et al*. [15, 61] for a more detailed description of the study site and the nest aggregations). During 2009 and 2010, wasp females were marked, weighed and monitored, and their prey obtained; also during these years, the environmental availability of dipterans was surveyed. In 2011, a number of prey and predators were obtained to calculate their *FMR* and *WL*. Some dipterans belonging to the species *Sphaerophoria scripta* (L., 1758) were collected to test for sex-based differences in the values of the flight-related morphological traits (see below).

### Sample collection

In 2009–2010, the nests of 24 females of *B*. *merceti* and 21 of *B*. *zonata* were located and monitored to obtain the dipterans, which were stolen from the wasps when they returned to their nests after provisioning flights. A total of 276 prey from *B*. *merceti* females and 212 from *B*. *zonata* ones were obtained; subsequently, they were weighed, killed by freezing, stored in vials, pinned and identified to species/morphospecies [15].

To evaluate the abundance of the fly species available in the environment, in 2009–2010 we performed 5-minute hourly surveys of dipterans in the surroundings of the nesting area (to a distance of 300 m from the centre of the nesting area, as this is the maximum distance where marked *Bembix* females have been previously recaptured [15]). These samplings were taken between 11:00 and 18:00 h, the time when *Bembix* females concentrate their hunting activity [15, 57], over 12–16 days, collecting all the observed fly individuals with an entomological net. One to four individuals of each species/morphospecies from this sample were frozen and pinned for determination, and the rest were identified *in situ* based on the previously determined specimens, weighed immediately (within 5–10 minutes after the end of each 5-minute sampling), and released. A total of 454 dipterans, spanning 10 families and 50 species, were sampled.

In 2011, one to four individuals of each of these 50 species/morphospecies (overall, 87 individuals), and 17 *Bembix* females (10 *B*. *merceti* and 7 *B*. *zonata*) were collected from the environment as they were encountered (capturing the first individuals encountered, without any kind of selection), using an entomological net, and used for the characterization of flight morphology (see below). Owing to limitations in our access to the necessary equipment to perform a correct processing of the insects for their morphological analysis (i.e. a freezer near the field to keep the samples in good conditions until their processing), the sample collection for this part of the study had to be restricted to 2011.

During the sampling, flies could not be sexed because of the generally scarce dimorphism in external morphology (to the observer’s eyes). However, a number of studies with insects (including Diptera) have detected sex-based variations in body size, *FMR* and *WL* [62–64]. Thus, we tested, from an independent sample of flies (15 females and 14 males) collected with an entomological net in a neighbouring site, the sexual dimorphism of *Sphaerophoria scripta* (with regard to their body size, thorax mass, wing area, *WL* and *FMR*), a species in which males and females are relatively easier to distinguish, and the species far more represented (>60%) in both the environment and among the prey of both predator species, and in both 2009 and 2010 (see Results). We were unable to repeat the same control for all the prey species, but we think that, owing to the extreme abundance of this species both in the environment and among the captures, it is possible that the trend shown by *S*. *scripta* is the one that prevails overall in the collected dipteran sample.

### Morphological analysis

Flies and wasps collected for the morphological analysis were first weighed to the nearest milligram. Then, their thoraces and wings were carefully withdrawn from the rest of the body, using a pair of sharp entomological tweezers, and processed separately. The thoraces were weighed, and the flight muscle mass was estimated as 95% of the thorax mass, the value empirically obtained by Marden [29] for both Diptera and Hymenoptera. The *FMR* was then calculated as the flight muscle mass divided by the total body mass of each individual. The wings, for their part, were glued to sheets of white paper, scanned at 400 ppi resolution, and the images analysed with the software ImageJ (National Institutes of Health of the USA), where the wing area was calculated. Only one wing per individual was processed in the case of the dipterans (left wing), and the two left wings were utilized in the case of the wasps, multiplying by two the output values to get the total wing surface for each individual. *WL* was calculated as the ratio of total body mass (g) to total wing area (cm^2^).

Using the flight muscle mass and the body mass, we estimated the theoretical maximum load (or maximum prey mass) that a wasp is able to carry in flight, by means of the regression equation of maximum lift force *versus* flight muscle mass for bees and wasps provided in Table 5 of Marden [29].

### Statistical analyses

In our sample of flies processed to obtain the flight-related parameters, we calculated the mean *WL*, *FMR* and body mass for each of the species; then we transformed *WL* into their natural logarithm to achieve normality, and conducted linear regressions to assess the potential linear relationships between the species’ body mass and *FMR*, between their body mass and *WL*, and between their *FMR* and *WL*. Because thorax mass and wing area can potentially scale allometrically with body mass, as it has previously been observed, in particular for *WL* (e.g, [38, 46, 65, 66]), we tested for allometric relationships with the Major Axis Regression method, which accounts for the variation in both variables, not only in the independent variable (body mass). This method calculates the slope of the log-log regressions, together with the 2.5% and 97.5% confidence intervals. If the predicted isometric slopes fall outside the confidence intervals, the dependent variables scales allometrically with the independent variable, otherwise the relationships are isometric. These tests were performed with the software R 3.2.3 (lmodel2 package).

We carried out ANCOVAs (one per each wasp species), with a manual stepwise backwards procedure, where only the variables with *P*<0.05 (or those taking part in a significant interaction) were kept in the model, to determine the factors involved in the lesser or greater predation frequency of the different prey species, where the response variable was the capture frequency of the different prey species (square-root transformed to achieve normality). We initially included as explanatory variables “environmental availability” (i.e. the abundance of a fly species in the environment, expressed as the absolute number of individuals belonging to this species in each of the years, square-root transformed for the sake of normality), “*FMR*” (mean value per species), “*WL*” (mean value per species), “year” and the interactions “environmental availability*FMR”, “environmental availability*WL”, “environmental availability*year”, “FMR*WL”, “FMR*year” and “WL*year”, and relied on the Type III ANOVA to decide which variables to keep in the model. ANCOVAs with a similar stepwise procedure were applied to our sample of 17 female wasps used to characterize flight morphology, in order to analyse the differences in *FMR*, *WL*, and wing area (fourth root-transformed to achieve normality) between wasp species, initially using as explanatory variables “wasp mass”, “wasp species” and “wasp mass*wasp species”. Possible linear relationships between the *FMR* and the *WL* of the wasps were investigated through a linear regression. ANOVAs were run to compare the effects on prey capture of the dipteran *FMR*, *WL* and environmental availability between the two years studied.

In our sample of 45 marked females, Student’s *t*-tests were used to look for differences of body mass between the females of the two *Bembix* species, and to compare the theoretical maximum load between both species of predators.

These statistics were carried out with XL STAT 2012 (Addinsoft).

The raw data used to perform these analyses are offered as Supporting Information (see S1 Dataset).

## Ethics statement

The necessary permits to perform the observation, manipulation and collection of insects were obtained yearly from the Junta de Castilla y León. The experiments performed for the development of this study obey the current Spanish law.

## Results

Prey of *B*. *merceti* and *B*. *zonata* consisted of Diptera belonging to the families Bombyliidae, Calliphoridae, Syrphidae, Sarcophagidae, Stratiomyidae, Tabanidae and Tachinidae. A total of 29 species/morphospecies were collected as prey, with the syrphid *Sphaerophoria scripta* being by far the most abundant prey species (representing 51.63–77.17% of the dipterans hunted by *B*. *merceti*, 34.23–69.84% of the dipterans captured by *B*. *zonata*, and 40.42–61.10% of those available in the environment, depending on the year). Males of *S*. *scripta* turned out to be slightly larger than females (Student’s *t*-test, *t*_19_ = 2.150, *P* = 0.045) and had greater thorax mass than females (Student’s *t*-test, *t*_18_ = 2.920, *P* = 0.009). However, males and females did not differ in terms of wing area (Student’s *t*-test, *t*_27_ = -1.500, *P* = 0.14) or *WL* (Student’s *t*-test, *t*_27_ = -0.71, *P* = 0.48), and only marginally differed in *FMR* (Student’s *t*-test, *t*_27_ = -1.99, *P* = 0.056). The extreme abundance of *S*. *scripta*, which lacks strong sexual dimorphism in flight morphology, suggests the potential effect of any male-female differences is reasonably weak in the whole sample.

The mean mass of the different dipteran species varied between 7.6 and 296 mg (Table 1). Both thorax mass (R^2^ = 0.92, P < 0.0001) and wing area (R^2^ = 0.76, *P* < 0.0001) increased with increasing body mass. Perfect isometry occurs with a slope of 1 for mass-mass relationships and 0.6666667 for mass-area relationships. Our analysis showed a slope for wing area of 0.74 and a confidence interval of 0.65–0.83, which includes the predicted value for isometry. On the hand, for thorax mass the slope was 1.11 and the interval was 1.05–1.18, so that the predicted isometric slope of 1 was just below the lower confidence limit, indicating an extremely weak allometric relationship. Mean *FMR* values for the different dipteran species ranged between 0.136 and 0.597 (Table 1), and mean *WL* for the different dipteran species varied between 0.026 and 0.194 g/cm^2^ (Table 1). *FMR* and *WL* were not correlated across fly species (linear regression, R^2^adj. = -0.021, *P* = 0.976). Dipteran *FMR* weakly and positively depended on body mass (linear regression, R^2^adj. = 0.033, *P* = 0.051); in contrast, *WL* of the flies strongly and positively depended on body mass (linear regression, R^2^adj. = 0.219, *P*<0.0001).

**Table 1:** Mean values of *WL* and *FMR* (±SD) for the different species/morphospecies of dipterans present in the area.

Species	*N*	Family	Mean *WL* (g·cm^-2^)	Mean *FMR*	Mean mass (mg)	Captured by
*Amictus variegatus*	24	Bom	0.071 ± 0.000	0.136 ± 0.000	15.750 ± 2.250	*Bmer*
*Anthrax anthrax*	2	Bom	0.086 ± 0.000	0.543 ± 0.000	63.000 ± 0.000	nc
Asilidae_1	4	As	0.143 ± 0.033	0.351 ± 0.056	189.250 ± 0.000	nc
Asilidae_2	2	As	0.043 ± 0.000	0.351 ± 0.011	161.000 ± 0.000	nc
Asilidae_3	2	As	0.077 ± 0.000	0.291 ± 0.000	36.000 ± 0.000	nc
Asilidae_4	2	As	0.129 ± 0.000	0.346 ± 0.000	176.000 ± 0.000	nc
Asilidae_5	2	As	0.101 ± 0.000	0.310 ± 0.014	49.000 ± 0.000	nc
Asilidae_6	2	As	0.144 ± 0.000	0.487 ± 0.000	205.000 ± 0.000	nc
Bombyliidae_1	1	Bom	0.026 ± 0.000	0.356 ± 0.000	14.000 ± 0.000	nc
Bombyliidae_2	3	Bom	0.027 ± 0.000	0.380 ± 0.000	10.000 ± 0.000	nc
Bombyliidae_3	3	Bom	0.070 ± 0.000	0.438 ± 0.000	39.000 ± 0.000	nc
Bombyliidae_4	2	Bom	0.083 ± 0.000	0.498 ± 0.000	82.000 ± 0.000	nc
Bombyliidae_5	2	Bom	0.059 ± 0.000	0.411 ± 0.000	37.000 ± 0.000	nc
*Bombylius* sp.	2	Bom	0.119 ± 0.007	0.496 ± 0.017	58.000 ± 0.000	nc
*Calliphora vicina*	2	Calli	0.067 ± 0.012	0.471 ± 0.107	29.000 ± 0.000	nc
Calliphoridae_1	2	Calli	0.079 ± 0.000	0.342 ± 0.007	25.000 ± 0.000	nc
*Cerdistus erythrurus*	3	As	0.073 ± 0.011	0.269 ± 0.008	21.000 ± 0.000	*Bzon*
*Cheilosia* sp.	2	Syr	0.070 ± 0.013	0.396 ± 0.157	25.231 ± 0.000	*Bzon*
*Chrysops caecutiens*	3	Tab	0.031 ± 0.009	0.447 ± 0.072	32.000 ± 13.000	*Bmer*
*Cylindromyia* sp.	5	Tach	0.074 ± 0.012	0.369 ± 0.075	24.000 ± 0.000	nc
*Eupeodes corollae*	4	Syr	0.045 ± 0.001	0.498 ± 0.192	35.250 ± 11.750	nc
*Exhyalanthrax afer*	2	Bom	0.052 ± 0.001	0.211 ± 0.192	9.000 ± 0.000	*Bmer*
*Exoprosopa jacchus*	2	Bom	0.042 ± 0.000	0.317 ± 0.000	51.000 ± 0.000	nc
*Haematopota ocelligera*	3	Tab	0.056 ± 0.004	0.417 ± 0.047	28.000 ± 0.000	*Bmer; Bzon*
*Hemipenthes velutinus*	8	Bom	0.046 ± 0.010	0.423 ± 0.026	36.457 ± 2.257	*Bzon*
*Merodon nigritarsis*	2	Syr	0.120 ± 0.000	0.365 ± 0.000	50.750 ± 6.250	*Bmer*
Miltogramminae_1	6	Sar	0.094 ± 0.000	0.297 ± 0.000	16.500 ± 1.500	*Bmer; Bzon*
Miltogramminae_2	16	Sar	0.059 ± 0.026	0.541 ± 0.059	14.667 ± 0.333	nc
*Musca larvipara*	2	Mus	0.052 ± 0.000	0.475 ± 0.000	16.000 ± 0.000	nc
*Musca* sp.	1	Mus	0.073 ± 0.000	0.475 ± 0.000	20.000 ± 0.000	*Bmer*
*Odontomyia* sp.	59	Strat	0.194 ± 0.009	0.421 ± 0.074	12.989 ± 0.111	nc
*Paragus* sp._1	5	Syr	0.057 ± 0.013	0.252 ± 0.052	7.600 ± 0.000	nc
*Paragus* sp._2	5	Syr	0.029 ± 0.000	0.397 ± 0.146	5.600 ± 0.000	*Bmer; Bzon*
*Peleteria* sp.	12	Tach	0.135 ± 0.013	0.408 ± 0.075	64.076 ± 1.258	*Bmer; Bzon*
*Pollenia rudis*	12	Calli	0.049 ± 0.010	0.469 ± 0.095	21.000 ± 2.000	nc
Sarcophagidae_1	2	Sar	0.092 ± 0.008	0.519 ± 0.001	92.500 ± 0.000	nc
Sarcophagidae_2	10	Sar	0.046 ± 0.000	0.317 ± 0.000	33.500 ± 0.000	nc
Sarcophagidae_3	3	Sar	0.033 ± 0.000	0.570 ± 0.000	7.667 ± 0.000	*Bmer; Bzon*
*Sphaerophoria scripta*	418	Syr	0.045 ± 0.007	0.305 ± 0.091	10.899 ± 0.595	*Bmer; Bzon*
*Stomorhina lunata*	5	Calli	0.078 ± 0.017	0.414 ± 0.056	21.875 ± 3.125	nc
Syrphidae_1	5	Syr	0.068 ± 0.000	0.190 ± 0.000	4.400 ± 0.000	*Bmer*
*Systoechus gradatus*	24	Bom	0.044 ± 0.010	0.462 ± 0.152	13.975± 0.825	*Bmer; Bzon*
*Tabanus rectus*	1	Tab	0.096 ± 0.000	0.532 ± 0.000	296.000 ± 0.000	nc
Tachinidae_1	8	Tach	0.048 ± 0.022	0.597 ± 0.004	14.000 ± 0.000	nc
Tachinidae_2	8	Tach	0.044 ± 0.009	0.528 ± 0.125	21.167 ± 4.833	nc
Tephritidae_1	2	Teph	0.036 ± 0.011	0.396 ± 0.112	25.000 ± 0.000	*Bmer*
*Thyridanthrax elegans*	13	Bom	0.047 ± 0.007	0.387 ± 0.159	20.188 ± 8.188	nc
*Usia aenea*	27	Bom	0.092 ± 0.016	0.364 ± 0.097	20.739 ± 0.989	*Bmer; Bzon*
*Villa hottentotta*	16	Bom	0.094 ± 0.016	0.436 ± 0.081	71.500 ± 1.625	*Bmer; Bzon*
*Villa paniscus*	19	Bom	0.049 ± 0.000	0.475 ± 0.000	16.891 ± 0.291	*Bmer; Bzon*

It is also indicated if the species are captured by *B*. *merceti* (*Bmer*), *B*. *zonata* (*Bzon*) or not captured by any of the species (nc), as well as the sample size for each species. Key for the families of dipterans: As = Asilidae; Bom = Bombyliidae; Calli = Calliphoridae; Mus = Muscidae; Sar = Sarcophagidae; Strat = Stratiomyidae; Syr = Syrphidae, Tab = Tabanidae; Tach = Tachinidae; Teph = Tephritidae.

*B*. *zonata* females had greater mean body mass than those of *B*. *merceti* (Student’s *t*-test, *t*_43_ = 2.608, *P* = 0.012) (Table 2). Wasp wing area did not vary with body mass or between species (ANCOVA, F_2,14_ = 2.055, R^2^adj. = 0.165, *P* = 0.156; Type III ANOVA: F(mass) = 0.023, *P*(mass) = 0.882, F(wasp species) = 1.943, *P*(wasp species) = 0.187). Wasp *FMR* depended on the wasp species and not on body mass (ANCOVA, F_2,14_ = 29.312, R^2^adj. = 0.780, *P*<0.0001; Type III ANOVA: F(mass) = 2.104, *P*(mass) = 0.169, F(wasp species) = 51.018, *P*(wasp species)<0.0001), having a greater mean value in *B*. *zonata* (Table 2). On the other hand, wasp *WL* positively scaled with body mass, and was not different between the two *Bembix* species (ANCOVA, F_2,14_ = 12.238, R^2^adj. = 0.584, *P* = 0.0001; Type III ANOVA: F(mass) = 20.769, *P*(mass) = 0.000, F(wasp species) = 0.616, *P*(wasp species) = 0.446) (Table 2). There was no relationship between wasps’ *FMR* and *WL* (linear regression, F_1,15_ = 0.005, R^2^adj. = -0.066, *P* = 0.944). The maximum theoretical load was significantly higher in *B*. *zonata* than in *B*. *merceti* (Student’s *t*-test, *t*_43_ = 17.409, *P*<0.0001) (Table 2).

**Table 2:** Mean values (±SD) of different biometric parameters for the predatory wasps.

	*B*. *merceti*	*B*. *zonata*
Wing area (cm^2^)	0.515 ± 0.041	0.591 ± 0.138
*FMR*	0.306 ± 0.037	0.430 ± 0.032
*WL* (g·cm^-2^)	0.184 ± 0.051	0.214 ± 0.039
Body mass (g)	0.097 ± 0.018	0.108 ± 0.014
Maximum theoretical load (g)	0.055 ± 0.012	0.129 ± 0.017
Mean *WL* (g·cm^-2^) of the captured prey	0.056 ± 0.041	0.059 ± 0.039
Mean *FMR* of the captured prey	0.358 ± 0.270	0.365 ± 0.247

**Fig 1:**
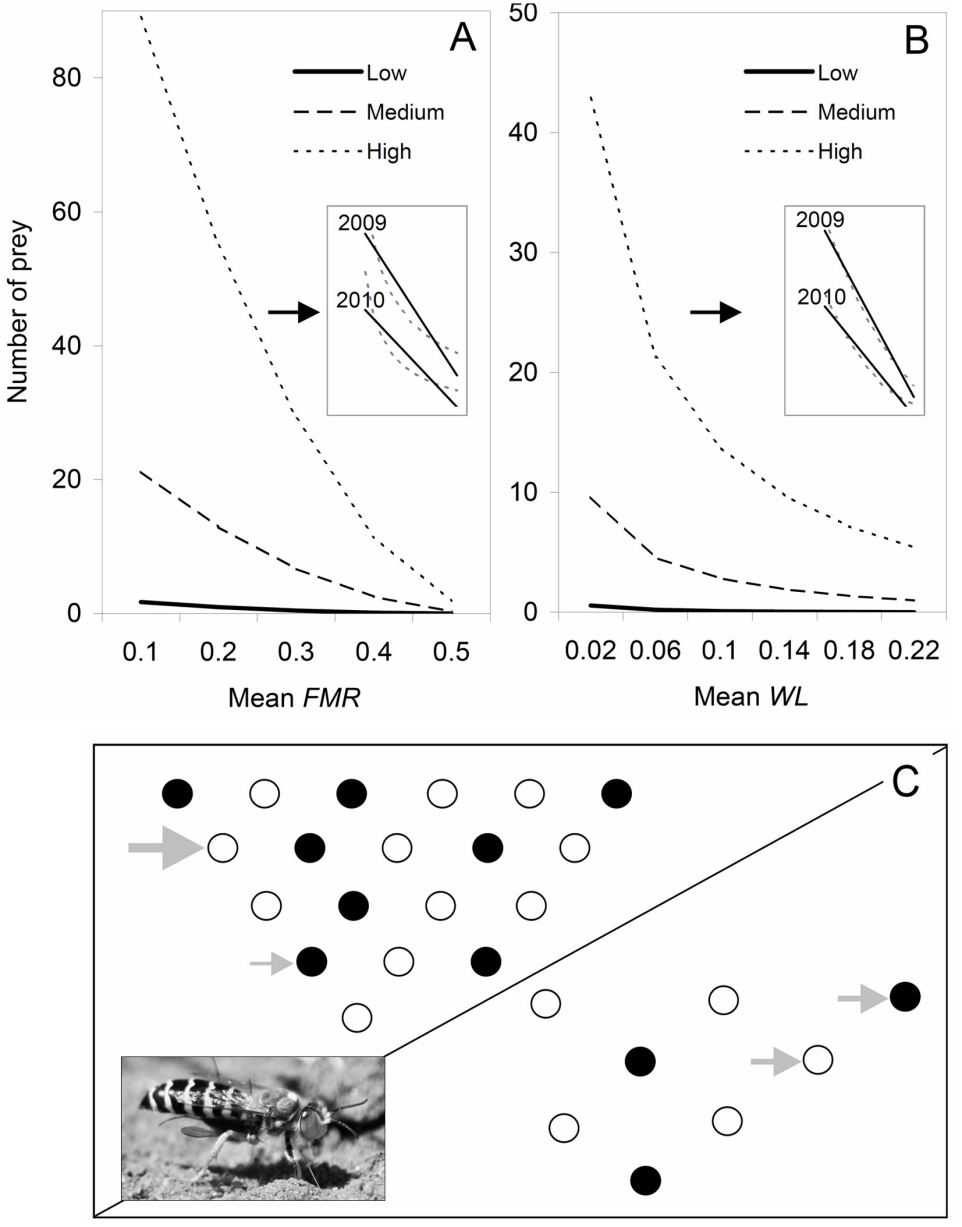
Effects of the interaction between prey availability and flight morphology on the predation by wasps. For *Bembix merceti* (A), the three lines show the relationship between flight muscle ratio (*FMR*) and the number of captured prey at three prey availability levels (low, medium and high) (2010 data). For *Bembix zonata* (B), the three lines show the relationship between wing loading (*WL*) and the number of captured prey at three prey availability levels (low, medium and high) (2010 data). Both (A) and (B) highlight that the effect of flight morphology on capture rate is stronger when prey are abundant in the environment. In both (A) and (B) the smaller inner box shows the lines for the highest level of prey abundance in both 2009 an 2010, together with their linear trend lines, in order to remark the greater influence of dipteran availability in 2009, the year of highest prey abundance (note the steeper slope). The same slope differences occurred also at other prey abundances (not shown). In (C), a representation of the wasps’ hunting behaviour in a situation of high (above the oblique line) or low (below the oblique line) prey availability is shown. When availability is high, wasps more often catch flies with lower *FMR* (white circles) (i.e. they are probably easier to hunt) and *WL* (white circles) (i.e. easier to hunt if reduced cruising speed is important) (large grey arrow) than those with great *FMR* and *WL* (black circles) (small grey arrow). When availability is low, resource scarcity limits avoidance of dipterans with better flight ability and wasps have to include more flies with greater *FMR* (i.e. which are likely more difficult to hunt) and *WL* (i.e. which are likely more difficult to hunt if improved cruising speed is important), so that both dipterans with low or high *FMR* and *WL* are equally hunted (identical medium-sized grey arrows). In (C), the picture in the bottom left corner shows a female *B*. *zonata* carrying its dipteran prey into the nest.

The ANCOVA (F_5,81_ = 32.135, R^2^adj. = 0.644, *P*<0.0001) showed that the capture rate of the different fly species in *B*. *merceti* is driven by the factors “environmental availability”, “environmental availability*FMR” and “environmental availability*year” (Table 3, Fig 1A). Thus, females of *B*. *merceti* hunted more often species with higher availability, and avoided prey with higher *FMR*, as long as prey abundance was high in the environment; also, females’ captures were more affected by dipteran availability in 2009 (comparison of the slopes: t = 3.084, *P* = 0.003). In *B*. *zonata*, on the other hand, only “environmental availability*WL” and “environmental availability*year” affected capture rate (F_5,81_ = 18.322, R^2^adj. = 0.502, *P*<0.0001) (Table 3, Fig 1B), with species with high *WL* being less abundantly hunted (as long as prey are abundant in the surroundings), and with greater influence of dipteran availability in 2009 (comparison of the slopes: t = 2.193, *P* = 0.031).

**Table 3:** Final models selected in a type III ANCOVA, with F and *P*-values of the factors affecting prey capture in *B*. *merceti* and *B*. *zonat*a.

***B*. *merceti***	**Sum of Squares**	**D.F.**	**F**	***P*-values**
environmental availability	26.182	1	27.537	<0.0001
*FMR*	2.682	1	2.821	0.097
year	0.327	1	0.344	0.559
environmental availability*FMR	10.905	1	11.470	0.001
environmental availability*year	9.040	1	9.508	0.003
***B*.*zonata***	**Sum of Squares**	**D.F.**	**F**	***P*-values**
environmental availability	3.174	1	2.945	0.090
*WL*	1.618	1	1.501	0.224
year	0.515	1	0.478	0.491
environmental availability*WL	8.604	1	7.985	0.006
environmental availability*year	5.183	1	4.810	0.031

## Discussion

Among the factors known to account for prey use in *Bembix* wasps, the availability of potential prey species in the environment and wasp body size undoubtedly play an important role, though there are probably additional variables that have not been studied to date [15]. Here, we tested whether some morphological traits directly related to flight ability of the potential prey and of wasps could also be involved in explaining why some dipteran species are widely hunted while others are not. In the following text, we discuss our results regarding the possible role of dipteran flight morphology on prey use, and the possible role of wasp flight morphology on the inter-specific differences in prey use.

Our analysis showed contrasting patterns of the effect of dipteran morphology on the capture rate by *Bembix* wasps. In one of the wasp species (*B*. *merceti*), prey capture was negatively associated with the *FMR* of the prey, as predicted by our starting hypothesis; on the contrary, in the other wasp species (*B*. *zonata*), prey capture was negatively associated with the *WL* of the prey, in disagreement with our prediction. In any case, prey flight morphology significantly influenced the prey-predator output. Furthermore, dipteran morphological traits were observed to have a role in prey capture particularly under certain conditions. Wasp flight morphology, on its part, could also help to explain the above-mentioned inter-specific contrasting results.

First, we found that capture rate is explained mainly by the availability, either alone (*B*. *merceti*), or through the interaction with *WL* (*B*. *zonata*), in such a way that dipteran species that were more abundant in the environment were captured more often by wasps. The high environmental availability of the dipterans per se (i.e. without taking part in an interaction) is only proportional to prey capture in *B*. *merceti*, and not in *B*. *zonata*. In the case of *B*. *merceti*, the availability of prey also modifies the effect of *FMR* on the frequency of capture (discussed below). In *B*. *zonata*, availability influences prey capture only through its interaction with *WL*. As the environmental availability is involved, either alone or as part of an interaction, in the lower or higher predation rate of the different dipterans, this factor can be considered essential in explaining the frequency of capture of the different prey species. Thus, a high abundance of the different prey species may be considered an important predation-risk factor for those species, though not necessarily for individuals, since this depends on the extent of the population in the environment, which could dilute individual risk. Previous studies with other prey-predator systems have already documented the important and positive effects of availability on the capture rate in wasps [67–70].

In our case, we found an additional interaction involving both the availability and the year, in both predator species: the effect an increase in prey availability had on the numbers of captures by wasps was stronger in 2009 (with respect to 2010), when the slope of the line representing the environmental availability against the capture frequency was steeper (Fig 1A and 1B). We found that two factors that could explain this result changed between the years. First, assuming that the number of *Bembix* marked females in each year is an estimate for wasp density, a higher wasp density existed in 2009 than in 2010 (72 *versus* 47 *Bembix* females). Second, if the number of environmental dipterans is employed as a proxy for prey availability (211 and 243 collected dipterans in 2009 and 2010, respectively), the average number of available prey per female was 2.93 in 2009 and 5.17 in 2010 (scarcer prey in 2009). This fact could have acted in unknown directions, for example, with a surplus of predators taking the whole range of available prey, thereby reducing the chance of the wasps choosing their preferred dipterans. Consequently, a given increase in prey availability in 2009 would have a much greater effect on the number of prey captures than any other year [71, 72].

Second, high values of *WL* were associated with a lower capture rate in one species (*B*. *zonata*), as long as they appeared together with high prey availability, with no significant effect of this morphological trait for the other *Bembix* species. As already observed in other insects, including Diptera and Hymenoptera [73–75], *WL* increased as a function of body mass in the studied dipterans, so that flies with higher *WL* also had greater mass. These bigger flies with higher *WL* may have greater flight speed [44, 46], so that higher values would effectively help to reduce the predation risk by *B*. *zonata*. The effect of interaction between prey availability and *WL* in *B*. *zonata* could derive from a situation in which wasp females cannot avoid catching dipterans with high *WL* under circumstances of low availability, and have to shift to flies which are more difficult to pursue (high *WL*) to maintain the foraging rate. Why the same trend has not been detected in *B*. *merceti*, despite its theoretically worse flight capacity (lower *FMR*), is a question that remains to be investigated. Speculation that the microhabitats where both predators hunt their prey are different, could be formulated to explain it: for example, Kalcounis and Brigham [75] observed that bats with greater *WL* foraged in areas with a low number of obstacles to detect and dodge (where high velocity is more important than a good manoeuvrability). In the same way, if *B*. *merceti* hunted in cluttered microhabitats, then high prey *WL* (and hence speed) wouldn’t be important in predator avoidance, and wouldn’t affect prey capture, as seems to occur in our study. However, low *WL* improves other flight parameters, such as manoeuvrability and take-off acceleration [21, 27, 41, 43–45], so that high *WL* would not help reduce predation risk. Further experiments in which flight performance, flight morphology and possibly other parameters, such as wing beat frequency, are measured at the same time in flies are needed, and in any case the effect of *WL* could be heterogeneous in predatory wasps, given that a certain relationship between this parameter and the capture rate has been found only in *B*. *zonata*.

On their part, because wasp *WL* varied only with body mass in the studied wasps, and did not differ between species, this trait is probably weakly involved in explaining why prey capture by each wasp species is determined by a different morphological trait of the dipterans.

Third, an effect of prey *FMR* on the capture rate of *B*. *merceti* was detected, with predators capturing less often prey with higher *FMR*, as long as prey are at the same time strongly available in the environment. This is in accordance with our hypothesis, as prey with higher *FMR* would be able to dodge obstacles more easily during flight in cluttered habitats [36, 39–42], and fly more quickly [29]. In agreement with previous studies with insects [29, 65], *FMR* and body mass only very weakly correlated in the studied dipterans. On the other hand, the largest wasp species, *B*. *zonata*, also had the greatest *FMR*. The comparatively better flier *B*. *zonata* may not be limited by the *FMR* of its prey and thus this factor was not significant in the model for this species, again in line with our hypothesis. Similarly to what has been suggested for dipteran *WL* in *B*. *zonata*, if *FMR* positively affects flight ability, the effect of the interaction between availability and *FMR* in *B*. *merceti* could match a scenario in which, under circumstances of low availability, female wasps cannot avoid dipterans with great *FMR*, which are more difficult to hunt.

Finally, it should be mentioned that the aim of this study has been the evaluation of the morphological prey traits possibly involved in prey capture. Nevertheless, factors not studied here, belonging to the prey (wing beat frequency, flight speed, age, health, odour, microhabitat inhabited, temporal overlap with the predators) or to the predators (e.g. foraging behaviour), could also play a role in prey capture, as described for other taxa, both invertebrates and vertebrates [3–16, 76, 77]. New studies addressing these points would help to offer a more complete view of the factors affecting prey capture in sand wasps.

## Supporting Information

S1 Dataset Rough matrices containing the data used to perform the analyses. (XLS)

## References

[1] Combes SA, Rundle DE, Iwasaki JM, Crall JD. Linking biomechanics and ecology through predator-prey interactions: flight performance of dragonflies and their prey. J Exp Biol. 2012; 215: 903–913. 10.1242/jeb.059394 22357584

[2] Dawkins R, Krebs JR. Arms races between and within species. P Roy Soc Lond B Bio. 1979; 205: 489–511. 10.1098/rspb.1979.0081 42057

[3] Alexander BA: Predator–prey interactions between the digger wasp *Clypeadon laticinctus* and the harvester ant *Pogonomyrmex occidentalis*. J Nat Hist. 1985; 19: 1139–1154.

[4] Norberg UM, Rayner JMV. Ecological morphology and flight in bats (Mammalia: Chiroptera): wing adaptations, flight performance, foraging strategy and echolocation. Phil Trans R Soc Lond G. 1987; 316: 335–427.

[5] Aldridge HDJN, Brigham RM. Load carrying and maneuverability in an insectivorous bat: test of the 5% “rule” of radiotelemetry. J Mammal 1988; 69: 379–382.

[6] Ehlinger TJ. Habitat choice and phenotype-limited feeding efficiency in bluegill: individual differences and trophic polymorphism. Ecology 1990; 71: 886–896.

[7] Stubblefield JW, Seger J, Wenzel JW, Heisler MM. Temporal, spatial, sex-ratio, and body-size heterogeneity of prey species taken by the beewolf *Philanthus sanbornii* (Hymenoptera: Sphecidae). Philos Trans R Soc B-Biol Sci. 1993; 339: 397–423. 10.1098/rstb.1993.0039 8098871

[8] Herzner G, Schmitt T, Linsenmair KE, Strohm E. Prey recognition by females of the European beewolf and ist potential for a sensory trap. Anim Behav. 2005; 70: 1411–1418.

[9] Polidori C, Boesi R, Isola F, Andrietti F. Provisioning patterns and choice of prey in the digger wasp *Cerceris arenaria* (Hymenoptera: Crabonidae): the role of prey size. Eur J Entomol. 2005; 102: 801–804.

[10] Polidori C, Boesi R, Pesarini C, Papadia C, Federici M, Bevacqua S, et al Temporal relationship between the prey spectrum and population structure of the weevil-hunting wasp *Cerceris arenaria* (Hymenoptera: Crabronidae). Zoological Studies. 2007a; 46: 83–91.

[11] Polidori C, Mendiola P, Asís JD, Tormos J, García MD, Selfa J. Predatory habits of the grasshopper-hunting wasp *Stizus continuus* (Hymenoptera: Crabronidae): diet preference, predator-prey size relationships and foraging capacity. J Nat Hist. 2009; 43: 2985–3000.

[12] Polidori C, Gobbi M, Chatenaud L, Santoro D, Montani O, Andrietti F. Taxon-biased diet preference in the “generalist” beetle-hunting wasp *Cerceris rubida* provides insights on the evolution of prey specialization in apoid wasps. Biol J Linn Soc. 2010; 99: 544–558.

[13] Polidori C, Santoro D, Asís JD, Tormos J. Individual prey specialization in wasps: predator size is a weak predictor of taxonomic niche width and niche overlap In: Polidori C, editor. Predation in the Hymenoptera: an evolutionary perspective. Kerala, India: Transworld Research Network; 2011 pp. 101–121.

[14] Coelho JR, Hastings J, Holliday CW, Mendell A. Load carriage during foraging in two species of solitary wasps. J Hym Res. 2008; 17: 57–63.

[15] Asís JD, Baños- Picón L, Tormos J, Ballesteros Y, Alonso M, Gayubo SF. Are solitary progressive-provisioning wasps optimal foragers? A study with the digger wasp *Bembix merceti* (Hymenoptera: Crabronidae). Behaviour. 2011; 148: 191–214.

[16] Coelho JR. Effects of prey size and load carriage on the evolution of foraging strategies in wasps In: Polidori C, editor. Predation in the Hymenoptera: an evolutionary perspective. Kerala, India: Transworld Research Network; 2011 pp. 23–38.

[17] Chai P, Srygley RB. Predation and the flight, morphology, and temperature of neotropical rain-forest butterflies. Am Nat. 1990; 135: 398–411.

[18] Domenici P. The scaling of locomotor performance in predator-prey encounters: from fish to killer whales. Comp Biochem Phys A. 2001; 131: 169–182. 10.1016/s1095-6433(01)00465-2 11733175

[19] Walker JA, Ghalambor CK, Griset OL, McKenney D, Reznick DN. Do faster starts increase the probability of evading predators? Funct Ecol. 2005; 19: 808–815.

[20] Lima SL. Predation risk and unpredictable feeding conditions: determinants of body mass in birds. Ecology. 1986; 67(2): 377–385.

[21] Pennycuick CJ. Bird flight performance. Oxford: Oxford University Press; 1989.

[22] Hedenström A. Flight performance in relation to fuel load in birds. J Theor Biol. 1992; 158(4): 535–537.

[23] Bednekoff PA, Houston AI. Avian daily foraging patterns: effects of digestive constraints and variability. Evol Ecol. 1994; 8(1): 36–52.

[24] Bednekoff PA. Translating mass dependent flight performance into predation risk: an extension of Metcalfe & Ure. Proc R Soc London B. 1996; 263: 887–889.

[25] Kullberg C, Fransson T, Jakobsson S. Impaired predator evasion in fat Blackcaps (*Sylvia atricapilla*). Proc R Soc Lond B Biol Sci.1996; 263: 1671–1675.

[26] Hedenström A, Rosén M. Predator versus prey: on aerial hunting and escape strategies in birds. Behav Ecol. 2001; 12: 150–156.

[27] Burns JG, Ydenberg RC. The effects of wing loading and gender on the escape flights of least sandpipers (*Calidris minutilla*) and western sandpipers (*Calidris mauri*). Behav Ecol Sociobiol. 2002; 52: 128–136.

[28] Aldridge HDJN. Turning flight of bats. J Exp Biol. 1987; 128: 419–425. 3559468 10.1242/jeb.128.1.419

[29] Marden JH. Maximum lift production during takeoff in flying animals. J Exp Biol. 1987; 130: 235–258.

[30] Lind J, Fransson T, Jakobsson S, Kullberg C. Reduced take-off ability in robins (*Erithacus rubecula*) due to migratory fuel load. Behav Ecol Sociobiol. 1999; 46: 65–70.

[31] Kullberg C, Jakobsson S, Fransson T. High migratory fuel loads impair predator evasion in sedge warblers. The Auk. 2000; 117: 1034–1038.

[32] Almbro M, Kullberg C. Impaired escape flight ability in butterflies due to low flight muscle ratio prior to hibernation. J Exp Biol. 2008; 211: 24–28. 18083728 10.1242/jeb.008219

[33] Almbro M, Kullberg C. The downfall of mating- the effect of mate-carrying and flight muscle ratio on the escape ability of a pierid butterfly. Behav Ecol Sociobiol. 2009; 63: 413–420.

[34] Almbro M, Kullberg C. Weight loading and reproductive status affect the flight performance of *Pieris napi* butterflies. J Insect Behav. 2012; 25: 441–452.

[35] Vogel S. Modes and scaling in aquatic locomotion. Integr Comp Biol. 2008; 6: 702–712. 10.1093/icb/icn014 21669826

[36] Srygley RB, Dudley R. Correlations of the position of center of body mass with butterfly escape tactics. J Exp Biol. 1993; 174: 155–166.

[37] Berwaerts K, Van Dyck H. Take-off performance under optimal and suboptimal thermal conditions in the butterfly *Pararge aegeria*. Oecologia. 2004; 141: 536–545. 15309609 10.1007/s00442-004-1661-9

[38] Polidori C, Nieves-Aldrey JL. Comparative flight morphology in queens of invasive and native Patagonian bumblebees (Hymenoptera: *Bombus*). C R Biol. 2015; 338: 126–133. 10.1016/j.crvi.2014.11.001 25499798

[39] Ellington CP. The aerodynamics of hovering insect flight. IV. Aerodynamic mechanisms. Philos T Roy Soc B. 1984; 305(1122): 79–113.

[40] Rayner JMV. Form and function in avian flight. Curr Ornithol. 1988; 5: 1–77.

[41] Marden JH, Chai P. Aerial predation and butterfly design: how palatability, mimicry, and the need for evasive flight constrain mass allocation. Am Nat. 1991; 138: 15–36.

[42] Srygley RB, Chai P. Flight morphology of Neotropical butterflies: palatability and distribution of mass to the thorax and abdomen. Oecologia. 1990; 84: 491–499. 28312965 10.1007/BF00328165

[43] Norberg UM. Wing design, flight performance, and habitat use in bats In: Wainwright PC, Reilly SM, editors. Ecological morphology: integrative organismal biology. University of Chicago Press; 1994 pp.205–239.

[44] Ellington CP. Limitations on animal flight performance. J Exp Biol. 1991; 160: 71–91.

[45] Dudley R. Biomechanics of flight in Neotropical butterflies: morphometrics and kinematics. J Exp Biol. 1990; 150: 37–53.

[46] Dudley R, Srygley RB. Flight physiology of Neotropical butterflies: allometry of airspeeds during natural free flight. J Exp Biol. 1994; 191:125–139. 9317473 10.1242/jeb.191.1.125

[47] Coelho JR, Hoagland J. Load-lifting capacities of three species of yellowjackets (*Vespula*) foraging on honey-bee corpses. Funct Ecol. 1995; 9: 171–174.

[48] Coelho JR, Hastings JM, Holliday CW, unpublished data.

[49] Hastings JM, Holliday CW, Coelho JR. Body size relationship between *Sphecius speciosus* (Hymenoptera: Crabronidae) and their prey: prey size determines wasp size. Florida Entomol. 2008; 91: 657–663.

[50] Coelho JR, LaDage LD. Foraging capacity of the great golden digger wasp *Sphex ichneumoneus*. Ecol. Entomol. 1999; 24: 480–483.

[51] Brockmann HJ. Provisioning behavior of the great golden digger wasp, *Sphex ichneumoneus* (L.) (Sphecidae). J. Kansas Entomol. Soc. 1985; 58: 631–655.

[52] Polidori C, Federici M, Trombino L, Barberini V, Barbieri V, Andrietti F. Weight, volume and unbalancing: loading constraints of mud dauber wasps carrying mud balls. J. Zool. 2009; 279: 187–194.

[53] Evans HE, O’Neill KM. The Sand Wasps: natural history and behaviour. Cambridge, MA: Harvard University Press; 2007.

[54] Bernard F. Observations sur les proies de quelques hyménoptères. Bull Soc Entomol Fr. 1934; 39: 247–250.

[55] Bernard F. Hyménoptêres prédateurs des environs de Fréjus. Ann Soc Entomol Fr. 1935; 104: 31–72.

[56] Asís JD, Gayubo SF, Tormos J. Data on the nesting behaviour of five European *Bembix* and description of the mature larvae of *B*. *merceti* and *B*. *rostrata* (Hymenoptera, Sphecidae). Deut Entomol Zschr. 1992; 39: 221–231.

[57] Asís JD, Tormos J, Gayubo SF. Nesting behaviour and provisioning in *Bembix merceti* and *Bembix zonata* (Hymenoptera: Crabronidae). J Nat Hist. 2004; 38: 1799–1809.

[58] Ballesteros Y, Tormos J, Gayubo SF, Asís JD. Notes on the prey, nesting behaviour and natural enemies of three *Bembix* sand wasps (Hymenoptera: Crabronidae) in the Iberian Peninsula. Ann Soc Entomol Fr. 2012; 48: 281–288.

[59] Ballesteros Y, Polidori C, Tormos J, Baños-Picón L, Asís JD. Complex-to-predict generational shift between nested and clustered organization of individual prey networks in digger wasps. PLoS ONE. 2014; 9(7): e102325 10.1371/journal.pone.0102325 25019164 PMC4096507

[60] Polidori C, Santoro D, Blüthgen N. Does prey mobility affect niche width and individual specialization in hunting wasps? A network-based analysis. Oikos. 2013b; 122: 385–394.

[61] Asís JD, Ballesteros Y, Tormos J, Baños-Picón L, Polidori C. Spatial nest-settlement decisions in digger wasps: conspecifics matter more than heterospecifics and previous experience. Ethology. 2014; 120(4): 340–353.

[62] Ahman M, Karlsson B. Flight endurance in relation to adult age in the green-veined white butterfly *Pieris napi*. Ecol Entomol. 2009; 34: 783–787.

[63] Marden JH. Bodybuilding dragonflies: costs and benefits of maximizing flight muscle. Physiol Zool. 1989; 62: 505–521.

[64] Gurevitz JM, Kitron U, Gürtler R. Flight muscle dimorphism and heterogeneity in flight initiation of field-collected *Triatoma infestans* (Hemiptera: Reduviidae). J Med Entomol. 2007; 44(2): 186–191. 17427685 10.1603/0022-2585(2007)44[186:fmdahi]2.0.co;2 PMC2254500

[65] Polidori C, Crottini A, Della Venezia L, Selfa J, Saino N, Rubolini D. Food load manipulation ability shapes flight morphology in females of central-place foraging Hymenoptera. Front Zool. 2013a; 10: 36. 23805850 10.1186/1742-9994-10-36 PMC3698194

[66] Riskin DK, Iriarte-Díaz J, Middleton KM, Breuer KS, Swartz SM. The effect of body size on the wing movements of pteropodid bats, with insights into thrust and lift production. J Exp Biol 2010; 213: 4110–4122. 10.1242/jeb.043091 21075953

[67] Griffiths D. Prey availability and the food of predators. Ecology. 1975; 56: 1209–1214.

[68] Belwood JJ, Fenton MB. Variation in the diet of *Myotis lucifugus* (Chiroptera: Vespertilionidae). Can J Zool. 1976; 54(10): 1674–1678.

[69] Swift SM, Racey PA, Avery MI. Feeding ecology of *Pipistrellus pipistrellus* (Chiroptera: Vespertilionidae) during pregnancy and lactation. II. Diet. J Anim Ecol. 1985; 54(1): 217–225.

[70] Rydell J. Foraging and diet of the Northern bat *Eptesicus nilssoni* in Sweden. Hol Ecol. 1986; 9(4): 272–276.

[71] Begon M, Townsend CR, Harper JL. Ecology: from individuals to ecosystems. Blackwell Publishing; 2006.

[72] Spataro T, Bacher S, Bersier LF, Arditi R. Ratio-dependent predation in a field experiment with wasps. Ecosphere 2012; 3(12):1–12.

[73] Casey TM, Joos BA. Morphometrics, conductance, thoracic temperature, and flight energetics of noctuid and geometrid moths. Physiol Zool. 1983; 56(2): 160–173

[74] Starmer WT, Wolf LL. Causes of variation in wing loading among *Drosophila* species. Biol J Linn Soc. 1989; 37(3): 247–261.

[75] Kalcounis M, Brigham RM. Intraspecific variation in wing loading affects habitat use by little brown bats (*Myotis lucifugus*). Can J Zool. 1995; 73: 89–95.

[76] Linehan JE, Gregory RS, Schneider DC. Predation risk of age-0 cod (*Gadus*) relative to depth and substrate in coastal waters. J Exp Mar Biol Ecol. 2001; 263: 25–44.

[77] Martín J, de Neve L, Polo V, Fargallo JA, Soler M. Health-dependent vulnerability to predation affects escape responses of unguarded chinstrap penguin chicks. Behav Ecol Sociobiol. 2006; 60(6): 778–784.

